# Effectiveness of outpatient financing reform from health savings accounts to pooling scheme in China: a systematic review

**DOI:** 10.1080/16549716.2026.2750656

**Published:** 2026-10-23

**Authors:** Linjing Gong, Daopeng Duan, Shiju Dong, Jiajia Li

**Affiliations:** a Department of Social Medicine and Health Management, School of Public Health, Cheeloo College of Medicine, Shandong University, Jinan, China; b NHC Key Lab of Health Economics and Policy Research (Shandong University), Jinan, China; c Center for Health Management and Policy Research, Shandong University (Shandong Provincial Key New Think Tank), Jinan, China

**Keywords:** Outpatient financing reform, health savings accounts, pooling scheme, China, systematic review

## Abstract

Outpatient services are fundamental to safeguarding population health and promoting the efficient use of healthcare resources. However, China has long faced limited risk pooling and inadequate financial protection for outpatient services. In 2021, China launched a nationwide outpatient financing reform to address these limitations. This study examines the effectiveness of this reform, which shifted outpatient financing from individual health savings accounts (HSAs) to an outpatient pooling scheme. A systematic search of PubMed, Web of Science, and Chinese National Knowledge Infrastructure (CNKI) was conducted for studies published between 1998 and December 2025. A total of 21 studies were included in this review. Overall, the reform was generally associated with increased outpatient utilization. Several studies suggested substitution between outpatient and inpatient care, whereas others indicated complementary effects. Findings on medical expenditure and out-of-pocket spending were mixed, while limited evidence suggested increased primary healthcare utilization and improved health-related behaviors. Overall, the effects varied across outcomes and policy settings, highlighting the importance of policy design and local healthcare contexts.

## Paper Context

- **Main findings:** The shift from individual health savings accounts to pooled outpatient financing in China was generally associated with increased outpatient service utilization and, in some settings, reduced inpatient utilization and unnecessary hospitalizations, while evidence regarding patients’ financial burden and health insurance fund expenditures was mixed and limited evidence suggested greater use of primary healthcare facilities and healthier lifestyles.
- **Added knowledge:** This systematic review synthesizes the available empirical evidence and interprets the findings in terms of the potential incentive, substitution, compensatory, anti-poverty, spillover, and triage effects of China’s outpatient financing reform.
- **Global health impact for policy and action:** China’s reform experience may offer useful insights for countries seeking to improve healthcare resource allocation and strengthen outpatient financial protection, although differences in benefit design, baseline coverage, and health-system context should be carefully considered when adapting such reforms to other settings.

## Background

Outpatient services constitute a cornerstone of contemporary healthcare systems, playing a pivotal role in safeguarding population health and ensuring the efficient functioning of medical resources [1]. As the primary interface for disease prevention, early detection, and chronic condition management – including essential follow-up care such as postoperative rehabilitation – these services are indispensable for maintaining health system sustainability [2]. However, insufficient provision of outpatient care disrupts this equilibrium, compelling patients to resort to inpatient services unnecessarily [3]. This phenomenon perpetuates a detrimental cycle of low-value hospitalizations, where healthcare resources are misallocated, and patient outcomes are compromised [4,5].

Within this context, China’s healthcare reforms since the late 20th century have sought to reconcile the dual imperatives of equity and efficiency in service delivery for its 1.4 billion citizens [6]. A key component of these reforms has been the continued development of the Urban Employee Basic Medical Insurance (UEBMI) system, which was established in 1998. UEBMI functions through compulsory payroll-based contributions from both employers and employees, mandating the creation of the social pooling account (SPA) and individual health savings accounts (HSAs) to pay medical expenses, where the HSAs primarily cover outpatient care expenditures, while the SPA is designated for inpatient care and provides coinsurance support for inpatients [7,8]. During the early stages of development, China’s social health insurance (SHI) system prioritized the use of pooled funds for inpatient care, while providing insufficient financial protection for outpatient services. When general outpatient expenditure exceeded the available balance in their HSAs, patients were generally not eligible for additional reimbursement [9]. This arrangement may have distorted healthcare utilization in two ways: on the one hand, patients experiencing illness may have forgone or delayed outpatient care because of high out-of-pocket costs; on the other hand, it may have encouraged potentially avoidable hospital admissions [10]. Meanwhile, the limited risk-pooling capacity of HSAs contributed to both inadequate outpatient coverage and substantial fund accumulation. By the end of 2021, the aggregate balance of employees’ individual accounts had reached RMB 1.15 trillion, equivalent to more than RMB 3,200 per enrollee [11], suggesting substantial fund accumulation, limited liquidity, and suboptimal allocation under the existing regulatory framework [12,13].

In response to these systemic challenges, the Chinese government established a nationwide framework for UEBMI outpatient financing reform in April 2021. The reform revised the financing of HSAs by allocating all employer contributions to the social pooling fund, rather than crediting a portion of them to HSAs as under the previous arrangement, thereby expanding pooled financing and risk sharing for outpatient care [14]. Before the nationwide reform, however, various localities had already experimented with different forms of outpatient pooling and reimbursement [9,15], while outpatient pooling arrangements had also been developed for enrollees of resident health insurance schemes in some areas [16,17]. The 2021 reform established a nationwide policy framework to further strengthen pooled-fund protection, gradually expanding coverage to high-frequency and clinically significant outpatient services and setting the reimbursement rate for eligible general outpatient expenditures at no less than 50% [14].

China’s outpatient financing reform, including the restructuring of HSAs and expansion of outpatient pooling, represents a broader shift from reliance primarily on individual accounts to reimbursement through pooled funds [18]. The reform may affect not only healthcare utilization and expenditures but also care-seeking patterns, financial protection, resource allocation, and broader health-system performance [15,19]. Figure 1 summarizes the theoretical pathways through which China’s outpatient financing reform may influence these outcomes. China’s outpatient financing reform aims to optimize healthcare delivery by strategically redistributing medical resources and bolstering financial sustainability [6]. The adjusted outpatient benefits, including decreased cost-sharing [6], may influence outpatient utilization and associated costs through a price incentive effect, whereby lower out-of-pocket costs alter patients’ demand for outpatient care [20]. Improved access to outpatient services may also generate a substitution effect by replacing hospitalizations and reducing related inpatient expenditures [21]. However, outpatient and inpatient services may also exhibit complementary effects, whereby increased outpatient utilization may facilitate disease detection and referral, thereby increasing the use of necessary inpatient care [22].

**Figure 1.**
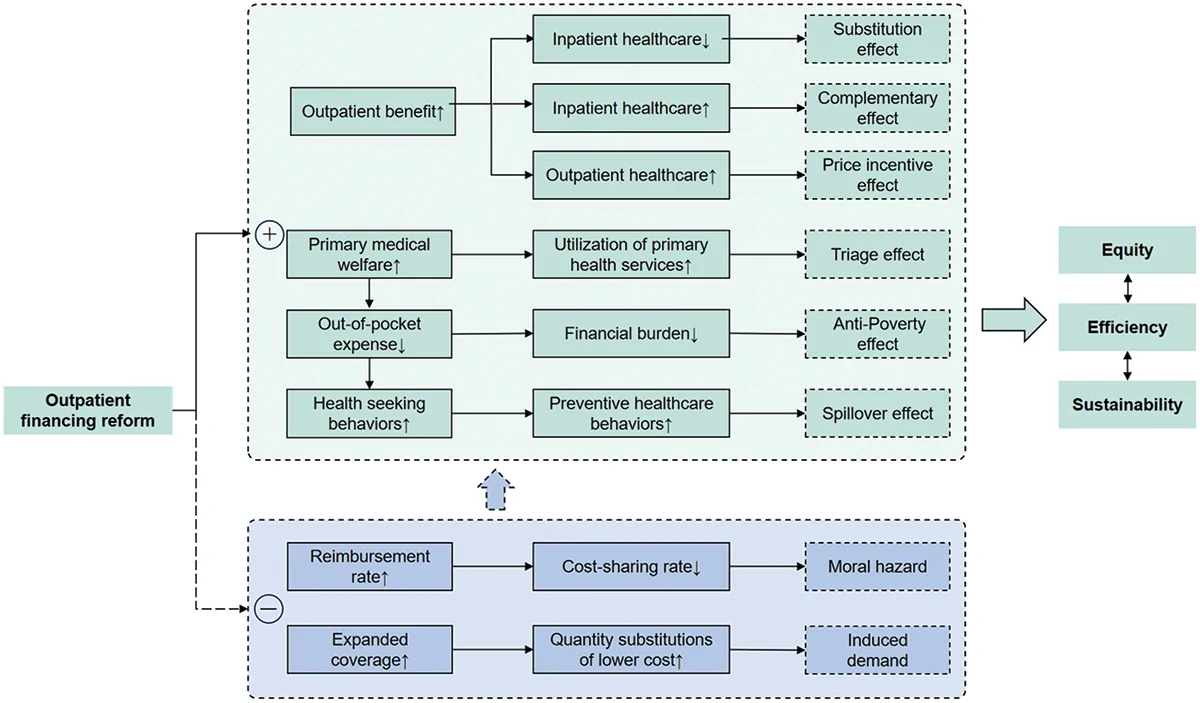
The mechanism of China’s outpatient financing reform from health savings accounts (HSAs) to pooling scheme.

Furthermore, outpatient financing reform may generate a patient-triage effect by providing more favorable reimbursement at primary healthcare institutions, thereby encouraging some outpatient visits to shift from secondary and tertiary hospitals to primary care and supporting tiered healthcare delivery [23]. The augmentation of outpatient and primary healthcare benefits enables enrollees to use outpatient services with reduced out-of-pocket expenses, thereby reducing their financial burden and mitigating the risk of poverty among economically disadvantaged individuals, thereby contributing to an anti-poverty effect [24]. Additionally, these outpatient incentive measures elevate the likelihood of patients seeking medical attention, increasing their exposure to health information and subsequently influencing their preventive healthcare behaviors, constituting a positive spillover effect [25]. However, an increase in the proportion of outpatient benefits and reimbursement may give rise to unnecessary treatment and examinations and potentially delay necessary hospitalization among severely ill patients, posing moral hazard and induced demand risks that necessitate prevention during the reform’s implementation [26–28].

However, existing systematic reviews have primarily examined the overall effects of health insurance coverage and related reforms on access to and utilization of healthcare, healthcare expenditure, financial protection, and health outcomes [29,30]. More specifically, some systematic reviews have examined payment methods for outpatient healthcare facilities and providers [31,32], as well as selected aspects of benefit design, such as patient cost-sharing [33,34]. However, these reviews have largely focused on supply-side payment incentives or specific services such as prescription drugs, with less attention to patient-side reforms to outpatient financing and benefit coverage. To our knowledge, the effects of China’s transition from HSAs to a pooled outpatient financing mechanism have not yet been specifically synthesized. Meanwhile, a growing body of empirical research has evaluated China’s outpatient mutual-aid financing reform, particularly its associations with healthcare utilization and financial protection [19,35]. However, individual studies have typically focused on specific outcomes, and their findings have not yet been systematically integrated.

Therefore, this systematic review synthesizes the available empirical evidence on China’s outpatient mutual-aid financing reform, with a particular focus on its associations with healthcare utilization, healthcare expenditure, financial protection, and other relevant outcomes. To elucidate the potential framework underlying these outcomes, the evidence is further examined through the lenses of price-incentive, substitution and complementarity, poverty-alleviation, patient-triage, and spillover effects. It also assesses the consistency and heterogeneity of findings across studies and outcomes and identifies remaining evidence gaps. The findings may inform the continued refinement of China’s health insurance system and provide context-specific insights for other settings seeking to design and evaluate outpatient benefit reforms while expanding financial protection and advancing equitable, efficient, and sustainable universal health coverage.

## Method

We designed the review process using the Synthesis Without Meta-analysis (SWiM) reporting guideline for narrative evidence synthesis [36]. This study adheres to the Preferred Reporting Items for Systematic Reviews and Meta-Analysis (PRISMA) standards [37]. The research question was formulated using the Population, Intervention, Comparison, Outcomes, and Study design (PICOS) framework [38].

## Inclusion and exclusion criteria

Based on the study objectives and the PICOS framework, the inclusion and exclusion criteria for study selection are presented in Table 1. The study population was limited to Chinese patients and healthcare institutions affected by or participating in the reform. Studies from other countries or regions were not considered. Eligible studies were required to evaluate the implementation or enhancement of outpatient financing reform as the intervention or exposure of interest. Studies in which outpatient financing reform was not the intervention or exposure of interest were excluded. Appropriate comparisons included pre-reform periods, populations not exposed to the reform, or other relevant comparison conditions. We considered outcomes related to patients, healthcare providers, and health systems, including healthcare utilization, medical expenditures, quality of care, patient satisfaction, financial protection, and healthcare delivery. Only longitudinal observational studies and experimental or quasi-experimental studies were included, while cross-sectional studies, qualitative research, narrative reports, and critical articles were excluded.

**Table 1.** Inclusion and exclusion criteria for study selection based on PICOS framework.

Outcome	Inclusion criteria	Exclusion criteria
Population	Chinese patients and healthcare institutions affected by or participating in the outpatient financing reform	Patients and healthcare facilities outside of China
Intervention	Implementation or enhancement of outpatient financing reforms	Studies in which outpatient financing reform was not the intervention or exposure of interest.
Comparator	Pre-reform periods, populations not exposed to the reform, or other appropriate comparison conditions	No controlled or poorly controlled studies
Outcome	Studies evaluating the effects of outpatient financing reform on healthcare utilization, healthcare expenditure, financial protection, or other relevant outcomes.	Studies that did not evaluate any outcomes related to outpatient financing reform.
Study design	Longitudinal observational studies (such as cohort studies) and experimental or quasi-experimental studies (such as randomized controlled trials and natural experiments)	Cross-sectional studies, qualitative research, narrative reports, critical articles

## Search strategy

Based on the research questions, a systematic literature search was created in PubMed. The search was also used on the Web of Science and Chinese National Knowledge Infrastructure (CNKI). The CNKI search was restricted to the Chinese Academic Journals database. Across all databases, eligibility was restricted to peer-reviewed journal articles. Grey literature, including master’s and doctoral dissertations, conference abstracts, preprints, and institutional or government reports, was not systematically searched and was excluded from the review. We used a combination of controlled words and keywords for the following concepts: (1) China (2) outpatients, (3) coordination, (4) medical insurance, (5) pooling, and (6) mutual aid. The complete database-specific search strategies are provided in Supplementary File 1. References to included studies and review articles were manually searched to identify any additional relevant studies for analysis. The search results were then reviewed. All titles and abstracts related to this study were searched as well as the full text. Literature search and data analysis were conducted from July 22 2026 to July 24 2026.

## Study selection

Selection for this study was conducted independently by two reviewers using predefined inclusion and exclusion criteria. All titles and abstracts identified through the search were independently screened by the two reviewers. All database records were imported into EndNote Reference software on 24 July 2026. The full texts of potentially eligible studies were subsequently retrieved and independently assessed by the same two reviewers. Any disagreements regarding study inclusion were resolved through discussion and consensus between the two reviewers. The screening process and results are reported in the study flow chart (Figure 2).

**Figure 2.**
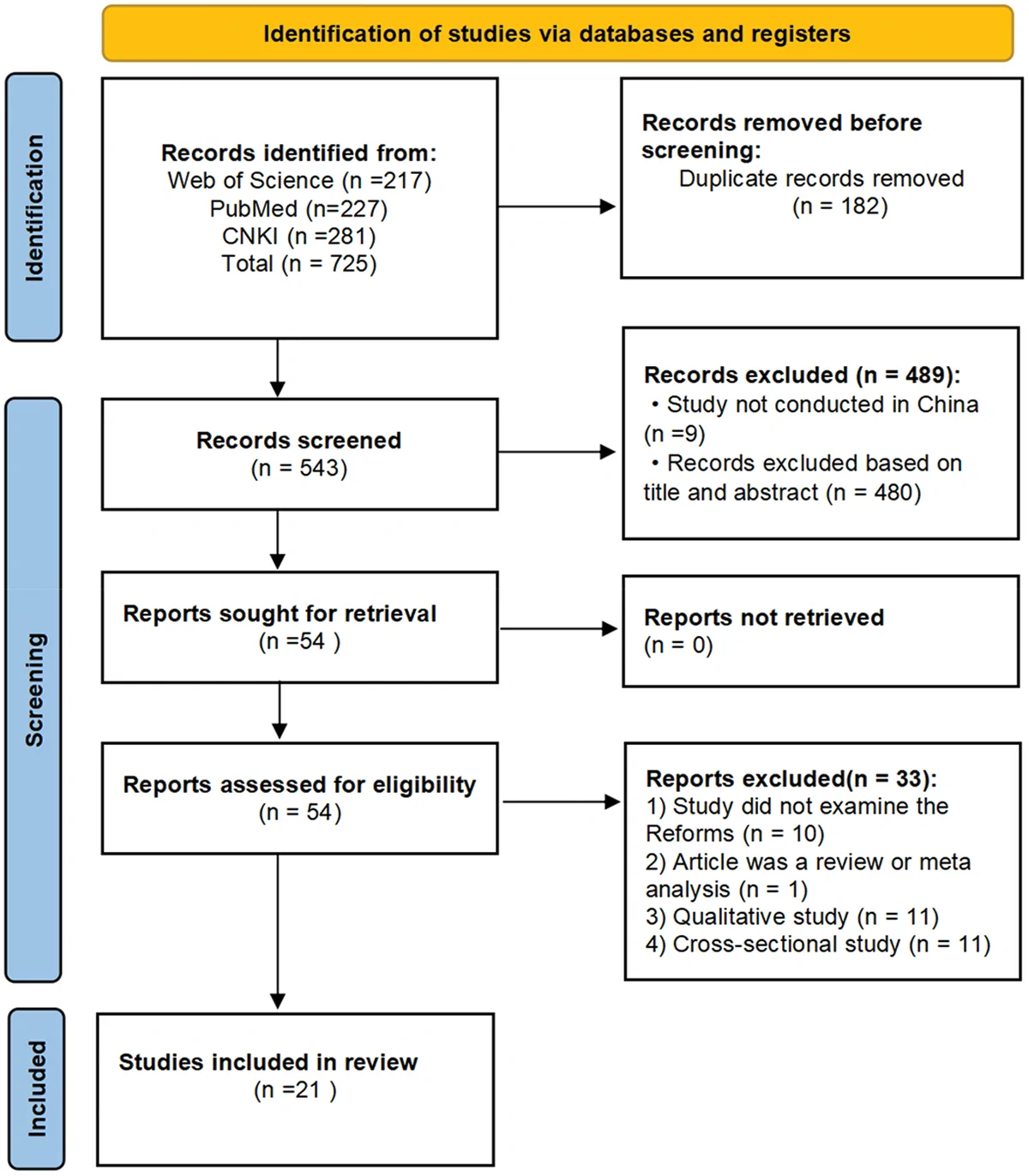
Preferred reporting items for systematic reviews and meta-analyses (PRISMA) flowchart of literature search and article selection process.

## Data extraction and quality assessment

By consulting similar review articles, two reviewers independently used Risk of Bias in non-randomized Studies of Interventions (ROBINS-I), developed by Sterne et al., to evaluate the risk of bias. ROBINS-I is a widely used risk-of-bias assessment tool for non-randomized studies of interventions. It requires the evaluator to respond to the signalling questions, including ‘Not applicable’, ‘Yes’, ‘Probably yes’, ‘Probably no’, ‘No’, and ‘No information’ [39]. Similar to the process of research selection, disagreements were resolved through discussion and consensus between the reviewers. The certainty of evidence was assessed separately for each outcome using the GRADE approach [40]. For non-randomized studies assessed using ROBINS-I, certainty was initially rated as high and then evaluated across five domains: risk of bias, inconsistency, indirectness, imprecision, and publication bias. Serious and very serious concerns resulted in downgrading by one and two levels, respectively. The final certainty was classified as high, moderate, low, or very low. Detailed assessment criteria and results for both ROBINS-I and GRADE are provided in Supplementary File 2.

## Synthesis

We provide a narrative description of the synthetic information. For studies using interrupted time-series (ITS) analysis, we reported the long-term post-intervention effects in the results tables to facilitate comparison across studies. Statistical analysis of the results was not conducted due to the different reporting methods of the included studies. Additionally, we grouped articles into different reform subgroups based on outcomes before and after the reform or intervention to synthesize the findings, and tabulated the data for better visualization. Findings were summarized according to the most commonly reported outcomes.

## Results

Our search identified 725 potentially eligible records, including 217 from Web of science, 227 from PubMed, and the remaining 281 from CNKI. After removing duplicate records, we screened 534 records and excluded 489 records for the reasons listed in Figure 2. In the full text review, we then assessed the eligibility of the remaining 54 studies and excluded 33 studies, also for the reasons shown in Figure 2. Data extraction and bias risk assessment were performed for 21 studies included in the systematic review. The characteristics of the included studies are summarized in Table 2. Of the 21 studies included, 18 examined the effects of outpatient financing reform on the utilization of outpatient and/or inpatient healthcare services. Several studies additionally examined health outcomes, health inequity, financial risk, or commercial medical insurance purchase. The publication dates spanned from 2014 to 2025. Thirteen studies focused on UEBMI enrollees, 2 focused on URRBMI enrollees, and 6 examined specific clinical populations, including patients with hypertension, diabetes, or cancer. Among the 21 studies, 20 employed quasi-experimental designs, while one used an observational study design. The quasi-experimental studies applied a range of analytical approaches, including difference-in-differences (DID), interrupted time series (ITS), regression discontinuity design (RDD), and propensity score matching (PSM), fixed-effects models, and segmented regression.

**Table 2.** Characteristics of studies included in the systematic review.

	Study			Population	Observation			Primary outcome
	**Source**	**Year**	**Data source**		**Duration**	**Study design**	**Analysis method**	
1	He, W. et al. [10]	2025	CHAD	Hypertension or diabetes patients	2015–2019	Quasi-experiment	Two-way fixed effects regression;	The utilization of outpatient and inpatient services
2	Jiang, J. et al. [11]	2025	CHAD	Oncology inpatients	2022.1–2023.7	Quasi-experiment	RDD regression	The utilization of inpatient services
3	Zhang, T. et al. [9]	2025	CHARLS	UEBMI enrollees aged ≥ 45	2011–2020	Quasi-experiment	PSM	The utilization inpatient services and health inequity
4	Du, W. et al. [35]	2022	CHAD	Diabetes patients	2019–2021	Quasi-experiment	Fixed effect specification	The utilization of outpatient, inpatient services and health outcomes
5	He, W [41].	2022	CHAD	UEBMI enrollees	2013–2014	Quasi-experiment	DID	The utilization of inpatient services
6	Wang, X. et al. [15]	2025	CHARLS	UEBMI enrollees	2011–2018	Quasi-experiment	DID	The utilization and inpatient services and health outconme
7	Wu, H. et al. [42]	2025	CHAD	UEBMI enrollees	2015.8—2018.8	Quasi-experiment	Segmented linear regression	The utilization of outpatient and inpatient services
8	Zhang T.et al. [43]	2025	CHNS	UEBMI enrollees	2000–2015	Quasi-experiment	DID	Financial risk outcomes
9	Chen M. et al. [44]	2025	CHAD	Hypertensive outpatients	2022.01–2023.12	Quasi-experiment	ITS	The utilization of outpatient services
10	Chen M. et al. [45]	2025	CHAD	Diabetic patients	2022.01–2023.12	Quasi-experiment	ITS	The utilization of outpatient services
11	Chen W. et al. [46]	2025	CHARLS	UEBMI enrollees	2011–2020	Quasi-experiment	DID	The utilization of medical services,health outcome, health inequity
12	Ding S. et al. [16]	2024	CHAD	URRBMI enrollees	2008—2021	Quasi-experiment	DID	The utilization of inpatient services
13	Duan X. et al. [23]	2025	CHARLS	UEBMI enrollees	2011–2018	Quasi-experiment	DID	The utilization of outpatient and inpatient services
14	Gao Y. et al. [47]	2014	CHAD	Hypertensive and/or diabetic patients	2009–2012	quasi-experimental design	Poisson, OLS	The utilization of outpatient
15	He W. et al. [17]	2022	CHAD	URRBMI enrollees	2013–2014	Quasi-experiment	DID	The utilization of outpatient and inpatient services
16	Hu C. et al. [48]	2025	Hospital administrative data	UEBMI enrollees	2022.01–2023.12	Quasi-experiment	ITS	The utilization of outpatient and inpatient services
17	Jiang P. et al. [49]	2024	CHARLS	UEBMI enrollees	2011–2020	Quasi-experiment	DID	Commercial Medical Insurance Purchase
18	Liu H. et al. [50]	2024	CHARLS	UEBMI enrollees	2011–2018	Quasi-experiment	DID	The utilization of medical services, and health outcome
19	Liu J. et al. [51]	2025	CHARLS	UEBMI enrollees	2011–2020	Quasi-experiment	DID	Health outcome
20	Wang Y. et al. [52]	2025	CHARLS	UEBMI enrollees	2018–2020	Observational study	Probit	The utilization of outpatient and inpatient services
21	Yang F. et al. [53]	2021	CHAD	UEBMI enrollees	2015.01–2021.02	Quasi-experiment	ITS	The utilization of outpatient and inpatient services

Note: CFPS: China Family Panel Studies.

CHAD: China’s health administration department.

Table 3 summarizes the findings from 13 of the 21 included studies (61.9%) that evaluated the incentive effects of China’s outpatient financing reform. Most studies reported increases in outpatient visits following the reform [9,10,15,35,48,52]. In contrast, Wu et al. [42], Chen W. et al. [46] and Yang et al. [53] found no significant change in the probability of outpatient visits. Overall, most studies reported increased outpatient utilization, although a small number found no significant effects.

**Table 3.** Results for outpatient’s incentive effect.

Outcome:	1	3	4	6	7	9	10	11	14	15	16	20	21
Outpatient visits	▲	▲	▲	▲	⊗			⊗			▲	▲	⊗
Total medical visits			▲										
Total medical expenditure	▲		▼							▼			
Average outpatient expenditure per visit	▲				▲			⊗			⊗		
Per capita outpatient expenditure	▲	*	▼	⊗					▲				
Total Reimbursement expenditure	▲		⊗		⊗					▼			⊗
Outpatient reimbursement expenditure			▲		▼	▼	▼						⊗
Total out-of-pocket expenditure	▲		▼							▼			
Outpatient out-of-pocket expenditure		▼		⊗	▲	▼	⊗		▲				
Total Reimbursement ratio	▲												
Outpatient Reimbursement ratio						▼	▼						
Day surgery cases											▲		

Note: ▲ = Significant increase in outcome of interest; ▼ = Significant decrease in outcome of interest; ⊗ = Non-significant result; * = Results vary among different entities or at different stages.

Findings regarding medical expenditure and patients’ financial burden were heterogeneous. Total medical expenditure increased in He W. et al. [10] but decreased in Du W. et al. [35] and He W. et al. [17]. Average outpatient expenditure per visit increased in He W. et al. [10] and Wu H. et al. [42], whereas Chen W. et al. [46] and Hu C. et al. [48] reported no significant changes. Per capita outpatient expenditure increased in He W. et al. [10] and Gao Y. et al. [47], decreased in Du W. et al. [35], showed no significant change in Wang X. et al. [15]. Zhang T. et al. reported a significant reduction only in 2013, with nonsignificant effects in the other survey waves [9]. Total reimbursement expenditure increased in He W. et al. [10], decreased in He W. et al. [17], and showed no significant changes in three studies [35,42,53]. Outpatient reimbursement expenditure increased in Du W. et al. [35], decreased in Wu H. et al. [42] and Chen M. et al. [44,45], and showed no significant change in Yang F. et al. [53]. Total out-of-pocket expenditure increased in He W. et al. [10] but decreased in Du W. et al. [35] and He W. et al. [17], while outpatient out-of-pocket expenditure showed mixed effects across studies. The total reimbursement ratio increased in He W. et al. [10], whereas the outpatient reimbursement ratio decreased in Chen M. et al. [44,45]. Overall, expenditure, reimbursement, and out-of-pocket outcomes showed considerable heterogeneity across studies.

As shown in Table 4, among the 21 included studies, 11 (52.4%) evaluated the substitution effects of China’s outpatient financing reform, primarily by examining changes in outpatient and inpatient service use and related expenditure. Four studies [10,15,35,52] reported increases in outpatient visits accompanied by reductions in inpatient admissions. Reductions in inpatient admissions were also reported by He W [41], Chen W. et al. [46], and He W. et al. [17]. Du W. et al. (Study 4) additionally reported an increase in total medical visits, while hospitalization days decreased in four studies [10,11,35,41]. He W [41]. and He W. et al. [17] reported significant reductions in hospitalizations for ambulatory care-sensitive conditions (ACSCs), while He W. et al. [17] also found fewer non-severe admissions (HCC = 0) and a lower HCC score. However, the substitution effect was not consistently observed. Hu C. et al. [48] reported increases in both outpatient and inpatient service use, whereas Wu H. et al. [42] and Yang F. et al. [53] found no significant changes in either outpatient visits or inpatient admissions.

**Table 4.** Results for price substitution and complementary effect.

Outcome:	1	2	4	5	6	7	11	15	16	20	21
Outpatient visits	▲		▲		▲	⊗	⊗		▲	▲	⊗
Inpatient admissions	▼		▼	▼	▼	⊗	▼	▼	▲	▼	⊗
Total medical visits			▲								
Hospitalization days	▼	▼	▼	▼							
Average inpatient expenditure per admissions	▼	▼		▼		⊗	▼	⊗	⊗		
Per capita inpatient expenditure	▼		▼	▼	⊗			▼			
Average outpatient expenditure per visit	▲					▲	⊗		⊗		
Per capita outpatient expenditure	▲		▼		⊗						
Total medical expenditure	▲		▼					▼			
Total Reimbursement expenditure	▲		⊗			⊗		▼			⊗
Outpatient reimbursement expenditure			▲			▼					⊗
Inpatient reimbursement expenditure		▼	▼	▼		⊗		▼			⊗
Inpatient Reimbursement ratio				⊗							
Total out-of-pocket expenditure	▲		▼					▼			
Outpatient out-of-pocket expenditure					⊗	▲					
Inpatient out-of-pocket expenditure				▼	⊗	⊗		▼			
Inpatient out-of-pocket ratio		⊗						⊗			
Ambulatory Care Sensitive Conditions, ACSCs				▼				▼			
Hierarchical Condition Category, HCC								▼			

Note: ▲ = Significant increase in outcome of interest; ▼ = Significant decrease in outcome of interest; ⊗ = Non - significant result.

Expenditure-related findings were more complex. Reductions in average inpatient expenditure per admission were reported in four studies [10,11,41,46], whereas three studies [17,42,48] reported no significant changes. Per capita inpatient expenditure decreased in four studies [10,17,35,41] but showed no significant change in Wang X. et al. [15]. Total medical expenditure increased in He W. et al. [10] but decreased in Du W. et al. [35] and He W. et al. [17]. Total reimbursement expenditure increased in He W. et al. [10], decreased in He W. et al. [17], and showed no significant changes in three studies [35,42,53]. Four studies reported decreases in inpatient reimbursement expenditure [11,17,35,41], while Wu H. et al. [42] and Yang F. et al. [53] reported no significant changes. The inpatient reimbursement ratio showed no significant change in He W [41]. Total out-of-pocket expenditure increased in He W. et al. [10] but decreased in Du W. et al. [35] and He W. et al. [17]. Inpatient out-of-pocket expenditure decreased in He W [41]. and He W. et al. [17], while Wang X. et al. [15] and Wu H. et al. [42] found no significant changes. Overall, inpatient expenditure outcomes generally declined, whereas changes in total expenditure and financial burden were less consistent across studies.

As presented in Table 5, 12 studies (57.1%) assessed the anti-poverty and financial protection effects of China’s outpatient financing reform. Zhang T. et al. [43] reported significant reductions in both catastrophic health expenditure and impoverishing health expenditure. Zhang T. et al. [9] reported reductions in socioeconomic inequalities in outpatient utilization and expenditure, while Chen W. et al. [46] found a significant reduction in health inequality.

**Table 5.** Results for anti-poverty effect.

Outcome:	1	3	4	5	6	7	8	9	10	11	14	15
Catastrophic health expenditure							▼					
Impoverishing health expenditure							▼					
health inequality		▼								▼		
Out-of-pocket expenses	▲		▼									▼
Outpatient out-of-pocket expenditure		▼			⊗	▲		▼	⊗		▲	
Inpatient out-of-pocket expenditure				▼	⊗	⊗						▼
Outpatient out-of-pocket ratio											▼	

Note: ▲ = Significant increase in outcome of interest; ▼ = Significant decrease in outcome of interest; ⊗ = Non - significant result.

Findings for out-of-pocket expenditure were less consistent. Total out-of-pocket expenditure increased in He W. et al. [10] but decreased in Du et al. [35] and He W. et al. [17]. Outpatient out-of-pocket expenditure decreased in Zhang T. et al. [9] and Chen M. et al. [44], increased in Gao et al. [47] and Wu et al. [42], and showed no significant changes in Wang X. et al. [15] and Chen M. et al. [45]. Inpatient out-of-pocket expenditure decreased in He W [41] and He W. et al. [17], whereas Wang X. et al. [15] and Wu et al. [42] found no significant changes. Gao et al. [47] additionally reported a significant reduction in the outpatient out-of-pocket ratio. Overall, broader financial-protection outcomes improved, whereas findings for out-of-pocket expenditure were mixed.

Table 6 shows that 6 studies (28.6%) examined spillover effects beyond healthcare utilization and expenditure. Du et al. [35] reported significant reductions in both the number and severity of complications. Wang X. et al. [15] reported improved self-rated health and fewer ADL limitations. Liu H. et al. [50] found no significant changes in self-rated health, the number of chronic diseases, or ADL limitations within the first 4 years after reform, but significant improvements emerged 5–6 years after implementation. Liu J. et al. [51] reported improved mental health and fewer ADL limitations. Chen W. et al. [46] found increased physical activity but no significant changes in physical examinations, smoking, or drinking. Jiang P. et al. [49] reported an increase in commercial medical insurance purchases.

**Table 6.** Results for spillover effect.

Outcome:	4	6	11	17	18	19
Number of chronic diseases					*	
Self-rated health (SRH)		▲			*	
Mental health						▲
Number of ADL limitations		▼			*	▼
Number of complications	▼					
Complications Severity Index	▼					
Physical activity			▲			
Physical examination			⊗			
Smoking			⊗			
Alcohol consumption			⊗			
Commercial Medical Insurance Purchase				▲		

Note: ▲ = Significant increase in outcome of interest; ▼ = Significant decrease in outcome of interest; ⊗ = Non - significant result; * = Results vary at different stages.

As summarized in Tables 7 and 7 (33.3%) of the 21 included studies assessed the triage effects of China’s outpatient financing reform. Most studies indicated a shift in outpatient utilization toward primary care institutions. Five studies [16,23,46,47,53] reported increased primary care utilization, while Ding S. et al. [16], Duan X. et al. [23], and Yang F. et al. [53] also found reductions in outpatient utilization at higher-level hospitals. Zhang T. et al. [9] reported stage-specific effects, with primary care visits increasing significantly in 2013 and 2018 and hospital visits increasing significantly in 2011–2015. Regarding inpatient utilization, Ding S. et al. [16] found a shift in admissions toward primary care institutions, whereas He W. et al. [17] and Yang F. et al. [53] reported no significant changes at secondary and tertiary hospitals.

**Table 7.** Results for triage effect.

Outcome:	3	11	12	13	14	15	21
Outpatient visits at primary care institutions	*	▲	▲	▲	▲		▲
Outpatient visits at secondary and tertiary hospitals	*		▼	▼			▼
Inpatient visits at primary care institutions			▲				⊗
Impatient visits at secondary and tertiary hospitals			▼			⊗	⊗

Note: ▲ = Significant increase in outcome of interest; ▼ = Significant decrease in outcome of interest; ⊗ = Non - significant result; * = Results vary at different stages.

## Discussion

### Outpatient utilization: overall increases, but sustained effects depend on benefit design and population characteristics

Most evidence suggests that outpatient visit probability or frequency increased following outpatient financing reform [9,15,35,48,52]. This is consistent with the theory of demand for medical care [8]: outpatient reimbursement lowers the effective price faced by patients and facilitates the use of care that may previously have been deferred because of out-of-pocket costs. Outpatient financing reform may also generate broader spillover effects beyond healthcare utilization, influencing individuals’ health status, health-related behaviors, and subsequent healthcare and insurance decisions [15,35,49,51]. Improved access to outpatient care may be particularly important for patients with chronic conditions such as hypertension and diabetes, who require long-term medication and regular follow-up [10,35,44,45,47].

However, the effects of outpatient financing reform may differ depending on whether the policy establishes pooled reimbursement for general outpatient services for the first time or further optimizes an existing pooling scheme. Where outpatient pooling was introduced from a baseline of no pooled outpatient coverage, thereby providing a substantial new source of financial protection, significant reductions in inpatient out-of-pocket expenditure were observed [17,41]. By contrast, reforms that mainly reallocated individual account funds or optimized reimbursement within an existing pooling scheme tended to produce more limited or less sustained effects [42].

The generosity of benefit design may further shape the magnitude and persistence of policy effects. In Sanming, for example, outpatient utilization showed no significant long-term trend, possibly because the relatively high deductible of RMB 1,000 and the low annual payment limit of RMB 3,000 constrained continued use. Compared with the current national average deductible of approximately RMB 300.3 and annual payment limits of RMB 3,944.2 for active employees and RMB 4,288.6 for retirees [54], these benefit parameters provided relatively limited protection. Consistent with previous evidence that higher marginal out-of-pocket prices can reduce subsequent healthcare spending [55,56], patients with high healthcare needs may be more likely to exhaust their outpatient benefits early, potentially weakening sustained incentives for outpatient utilization.

Responses also varied across population groups and benefit types. Younger enrollees showed larger declines in outpatient utilization than retirees in one reform [57], while chronic disease outpatient use increased as general outpatient use declined in another [10]. These findings further suggest that reform effects are shaped by both benefit design and differences in healthcare needs across populations.

### Outpatient and inpatient care: substitution and complementarity may coexist

Increased outpatient utilization was frequently accompanied by lower inpatient utilization, suggesting that improved outpatient coverage may substitute for some inpatient care [10,15,35,52]. Further reductions in inpatient admissions, hospital stays, and hospitalizations for ACSCs support the possibility that better access to outpatient treatment and chronic disease management may reduce some inpatient demand [17,41].

However, outpatient and inpatient care may also be complementary. In Wuhan, outpatient visits, inpatient admissions, and day-surgery utilization all increased after the outpatient mutual-aid reform [48]. This pattern may partly reflect the interaction with concurrent policies promoting day surgery and reimbursement for preoperative outpatient examinations, while improved outpatient coverage may also facilitate screening and diagnosis and thereby identify conditions requiring subsequent inpatient treatment [22]. International evidence similarly suggests that lower outpatient cost sharing may reduce some emergency or avoidable hospitalizations while increasing diagnostic admissions [58]. Previously uninsured patients with chronic conditions have also been reported to increase both outpatient and inpatient utilization after gaining Medicare coverage [59].

Current evidence does not clearly distinguish whether changes in hospitalization arise from improved outpatient disease management, newly identified treatment needs, or other changes in healthcare-seeking behavior. Further research is needed to disentangle these mechanisms.

### Expenditure effects depend on spending patterns and demand- and supply-side responses

Improved outpatient coverage does not necessarily reduce total medical expenditure. Its net effect depends on the relative magnitude of additional outpatient spending and savings in inpatient care [35]. Where the increase in outpatient expenditure exceeded the reduction in inpatient expenditure, total medical expenditure increased despite evidence of substitution between outpatient and inpatient care [10]. Substitution therefore does not necessarily translate into overall cost savings.

Supply-side responses may further affect expenditure. Outpatient care in China continues to rely heavily on fee-for-service payment in many settings [60], and under fee-for-service payment and information asymmetry, providers may have incentives to increase the intensity of examinations, treatments, or prescriptions [27,48]. Despite more generous outpatient benefits, some studies reported increases in average outpatient expenditure per visit or per person, indicating that improved coverage does not necessarily translate into lower outpatient spending [10,42,47].

Demand-side responses also matter. Lower cost sharing may increase healthcare demand, but additional utilization may include both previously unmet necessary care and additional use induced by lower prices [26,50]. Consequently, higher reimbursement does not necessarily translate into lower absolute out-of-pocket spending. Absolute out-of-pocket expenditure increased despite improved reimbursement or lower out-of-pocket shares in some settings [10,47], while outpatient out-of-pocket expenditure per visit initially declined but subsequently increased over time in another setting [42].

Finally, several studies had observation periods of only approximately 1 to 2 years, and may therefore have captured mainly immediate or short-term responses rather than longer-term adjustments in patient behavior, provider responses, and chronic disease management [11,44,45,48].

## Strengths and limitations

This study has several strengths. To the best of our knowledge, it is the first systematic review to comprehensively synthesize empirical evidence on outpatient financing reform in China, covering a broad range of outcomes, including healthcare utilization, medical expenditure, health insurance fund expenditure, financial protection, health outcomes, and patterns of healthcare use. The review also included evidence from different regions, population groups, and reform models, allowing comparison of policy effects across different benefit designs and implementation contexts. In addition, systematic risk-of-bias assessment and certainty-of-evidence grading were conducted to provide a more rigorous appraisal of the reliability of the available evidence.

Several limitations should also be acknowledged. First, substantial heterogeneity existed across the included studies in reform design, study populations, outcome measures, and analytical methods, limiting direct comparability and quantitative synthesis of the findings. Second, the available evidence was dominated by observational and quasi-experimental studies rather than randomized controlled trials. Although some studies employed quasi-experimental methods to strengthen causal inference, residual confounding and other sources of bias could not be fully excluded. Consequently, the certainty of the evidence was generally rated as low or very low using the GRADE framework, and the findings should be interpreted with appropriate caution. Third, evidence on key mechanisms remains limited, particularly regarding substitution and complementarity between outpatient and inpatient care, behavioral responses of patients and providers, and the effects of specific benefit parameters on healthcare utilization and expenditure. Fourth, because of the substantial heterogeneity in study designs, outcomes, and effect measures, no meta-analysis or formal assessment of publication or reporting bias was conducted; therefore, the possibility of bias due to unpublished or selectively reported findings cannot be excluded. Finally, all included studies were conducted in China, where outpatient financing policies vary substantially across regions; therefore, the generalizability of the findings to other health insurance systems and healthcare settings may be limited.

## Conclusion

The systematic review presents several interesting findings. Firstly, outpatient financing reforms in China were generally associated with increased outpatient service utilization and, in some settings, showed the potential to reduce inpatient utilization and improve financial protection, while their effects on health insurance fund expenditure remained mixed. Second, limited evidence suggests that these reforms may encourage greater use of primary healthcare facilities and promote healthier lifestyles among patients.

Future research should prioritize high-quality quasi-experimental designs to distinguish effects across different policy contexts, adopt interdisciplinary frameworks to assess cross-sectoral consequences, and establish longitudinal monitoring systems to detect potential unintended effects and inequalities. Addressing these gaps could strengthen the evidence base for refining reform design and evaluating its transferability across settings, while helping to align outpatient financing reforms with broader health system goals of equity, efficiency, and sustainability.

## Supplementary Material

Supplementary File.docx

ZGHA-OpenScience form.docx

PRISMA 2020 checklist (1).docx

## Data Availability

All data analyzed in this systematic review were extracted from the published studies cited in the manuscript. Supporting materials for this study are publicly available on the Open Science Framework (OSF) at https://doi.org/10.17605/OSF.IO/NFC9A.

## Open scholarship

This article has earned the Center for Open Science badge for Open Materials. The materials are openly accessible at https://doi.org/10.17605/OSF.IO/NFC9A
