# Supplementary material for: Effectiveness of outpatient financing reform from health savings accounts to pooling scheme in China: a systematic review
Source: Glob Health Action. 2026 Oct 23;19(1):2750656. doi: 10.1080/16549716.2026.2750656 (PMC13700478; doi:10.1080/16549716.2026.2750656)
Supplement: Supplementary file 3 [file ZGHA_A_2750656_SM6750.docx]

**Supplementary File 1. Detailed Search Strategies for All Databases**

**Web of Science**

The search was conducted on July 24, 2026, and covered publications from 1998 to December 2025. No language or document-type filter was applied during the search. Across all databases, eligibility was restricted to peer-reviewed journal articles. Grey literature, including master’s and doctoral dissertations, conference abstracts, preprints, and institutional or government reports, was not systematically searched and was excluded from the review. The search strategy was:

TS=(outpatient* OR “out-patient*” OR “out patient*”) AND TS=(pooling OR coordinat* OR “co-ordination” OR “mutual aid” OR “mutual-aid” OR “mutual assistance” OR “cost sharing” OR “cost-sharing” OR coinsurance OR “co-insurance” OR copay* OR “co-payment” OR “co-payments” OR “out-of-pocket” OR “out of pocket” OR reimburs* OR “benefit package” OR “benefits package” OR “financing scheme” OR “financing schemes” OR “Flexible Spending Accounts” OR “Health Savings Accounts” OR “Medical Savings Accounts” OR “Health Savings Account” OR “personal medical account”) AND TS=(“medical insurance” OR “health insurance” OR “social health insurance” OR “basic medical insurance” OR “basic health insurance” OR “Urban Employee Basic Medical Insurance” OR “Employee Basic Medical Insurance” OR UEBMI) AND TS=(China OR Chinese)

**PubMed**

The search was conducted on July 24, 2026, and covered publications from 1998 to December 2025. No language or document-type filter was applied during the search. Across all databases, eligibility was restricted to peer-reviewed journal articles. Grey literature, including master’s and doctoral dissertations, conference abstracts, preprints, and institutional or government reports, was not systematically searched and was excluded from the review. The search strategy was:

(“Outpatients”[Mesh] OR outpatient*[tiab] OR “out-patient”[tiab] OR “out-patients”[tiab] OR “out patient”[tiab] OR “out patients”[tiab]) AND (“Cost Sharing”[Mesh] OR “Deductibles and Coinsurance”[Mesh] OR pooling[tiab] OR coordinat*[tiab] OR “co-ordination”[tiab] OR “mutual aid”[tiab] OR “mutual-aid”[tiab] OR “mutual assistance”[tiab] OR “cost sharing”[tiab] OR “cost-sharing”[tiab] OR coinsurance[tiab] OR “co-insurance”[tiab] OR copay*[tiab] OR “co-payment”[tiab] OR “co-payments”[tiab] OR “out-of-pocket”[tiab] OR “out of pocket”[tiab] OR reimburs*[tiab] OR “benefit package”[tiab] OR “benefits package”[tiab] OR “financing scheme”[tiab] OR “financing schemes”[tiab] OR “Flexible Spending Accounts”[tiab] OR “Health Savings Accounts”[tiab] OR “Medical Savings Accounts”[tiab] OR “Health Savings Account”[tiab] OR “personal medical account”[tiab]) AND (“Insurance, Health”[Mesh] OR “Insurance, Health, Reimbursement”[Mesh] OR “Medical Savings Accounts”[Mesh] OR “medical insurance”[tiab] OR “health insurance”[tiab] OR “social health insurance”[tiab] OR “basic medical insurance”[tiab] OR “basic health insurance”[tiab] OR “Urban Employee Basic Medical Insurance”[tiab] OR “Employee Basic Medical Insurance”[tiab] OR UEBMI[tiab]) AND (“China”[Mesh] OR China[tiab] OR Chinese[tiab])

**CNKI (Chinese)**

The search was conducted on July 22, 2026, and covered publications from 1998 to December 2025. The search was restricted to journal articles, with no additional language filter applied. Across all databases, eligibility was restricted to peer-reviewed journal articles. Grey literature, including master’s and doctoral dissertations, conference abstracts, preprints, and institutional or government reports, was not systematically searched and was excluded from the review. The search strategy was:

主题=（职工医保门诊共济 OR 职工医保门诊统筹 OR 个人账户改革） AND 主题=（影响 OR 效果 OR 实证）

English translation: Subject = (outpatient mutual aid under employee medical insurance OR outpatient pooling under employee medical insurance OR individual account reform) AND Subject = (impact OR effect OR empirical)

**Supplementary File 2. Detailed Search Strategies for All Databases**

**Supplementary File 2 Table 1. Criteria for Risk-of-Bias Assessment Using ROBINS-I**

| **Domain** | **Risk of bias** |
| --- | --- |
| **Confounding** | **·Low risk of bias：**Important confounders, secular trends and co-interventions were adequately controlled, and design-specific assumptions, such as parallel trends for DID or continuity for RDD, were well supported.  **·Moderate risk of bias：**Most important confounders were addressed, although some residual confounding remained possible.  **· Serious risk of bias：**Important confounders, concurrent policies or design assumptions were inadequately addressed, or the comparison group was poorly justified.  **·Critical risk of bias：**No credible counterfactual was established, and uncontrolled confounding made the effect estimate uninterpretable.  **·No information** |
| **Selection of participants** | **·Low risk of bias：**Eligibility and follow-up were defined before policy exposure, and selection was unrelated to both intervention status and outcome.  **·Moderate risk of bias：**Some post-intervention restrictions or exclusions occurred but were unlikely to materially affect the estimate.  **·Serious risk of bias：**Selection was based on post-intervention utilization, diagnosis, survival, enrollment or other outcome-related characteristics.  **·Critical risk of bias：**The analytical sample was largely determined by post-intervention outcomes or utilization, with no adequate correction.  **·No information** |
| **Classification of intervention** | **·Low risk of bias：**Policy exposure was clearly defined using objective information, and intervention and comparison groups were clearly distinguished.  **·Moderate risk of bias：**Classification was generally appropriate, but some uncertainty remained regarding timing, intensity or regional implementation.  **·Serious risk of bias：**Exposure status was unclear, substantially misclassified, affected by spillover, or mixed with other policies.  **·Critical risk of bias：**Intervention and comparison conditions could not be meaningfully distinguished.  **·No information** |
| **Deviations from intended intervention** | **·Low risk of bias：**Policy implementation and analysis were consistent with the intended effect, with no important contamination, switching or differential implementation.  **· Moderate risk of bias：**Some implementation variation, behavioral response or contamination occurred but was unlikely to materially affect the result.  **·Serious risk of bias：**Important deviations, contamination, switching or additional interventions were not adequately addressed.  **·Critical risk of bias：**Deviations or analytical problems were so extensive that the intended policy effect could not be estimated.  **·No information** |
| **Missing data** | **·Low risk of bias：**Data were complete or nearly complete, or missingness was appropriately addressed using valid methods.  **·Moderate risk of bias：**Missing data occurred but were unlikely to materially alter the estimate.  **·Serious risk of bias：**Missingness was substantial, imbalanced or related to both policy exposure and outcome, and was inadequately handled.  **·Critical risk of bias：**Missing data were so extensive or systematic that a credible estimate could not be obtained.  **·No information** |
| **Outcome measurement** | **·Low risk of bias：**Outcomes were measured using objective or validated methods that were comparable across groups and periods.  **·Moderate risk of bias：**Measurement had some limitations, but methods were consistent and errors were unlikely to differ by intervention status.  **·Serious risk of bias：**Definitions, coding or measurement differed across groups or periods, or outcome validity was limited.  **· Critical risk of bias：**Outcome measures were fundamentally invalid or incomparable.  **·No information** |
| **Selection of reported result** | **·Low risk of bias：**Outcomes and analyses were prespecified or transparently reported, with no evidence of selective reporting.  **·Moderate risk of bias：**No protocol was available, but reported analyses were consistent with the stated objectives and methods.  **·Serious risk of bias：**Results were likely selected from multiple outcomes, time points, subgroups or models based on significance or direction.  **·Critical risk of bias：**Selective reporting was so extensive that the reported estimate was not credible.  **·No information** |

**Supplementary File 2 Table 2. Risk-of-Bias Assessment of Included Studies Using ROBINS-I**

| **No.** | **Author** | **Confounding** | **Selection of participants** | **Classification of interventions** | **Deviations from intended interventions** | **Missing data** | **Measurement of outcomes** | **Selection of reported results** | **Overall risk of bias** |
| --- | --- | --- | --- | --- | --- | --- | --- | --- | --- |
| 1 | He, W. et al. | Serious | Moderate | Low | Low | Low | Low | Low | Serious |
| 2 | Jiang, J. et al. | Serious | Moderate | Low | Low | Low | Low | Low | Serious |
| 3 | Zhang, T. et al. | Moderate | Moderate | Low | Low | Low | Moderate | Low | Moderate |
| 4 | Du, W. et al. | Serious | Low | Low | Low | Low | Low | Low | Serious |
| 5 | He, W. | Moderate | Low | Low | Low | Low | Low | Low | Moderate |
| 6 | Wang, X. et al. | Moderate | Low | Low | Low | Moderate | Moderate | Low | Moderate |
| 7 | Wu, H. et al. | Moderate | Low | Low | Low | Low | Low | Low | Moderate |
| 8 | Zhang, T. et al. | Moderate | Low | Low | Low | Moderate | Moderate | Low | Moderate |
| 9 | Chen M. et al. | Serious | Moderate | Low | Low | Low | Low | Low | Serious |
| 10 | Chen M. et al. | Serious | Moderate | Low | Low | Low | Low | Low | Serious |
| 11 | Chen W. et al. | Moderate | Low | Low | Low | Low | Low | Moderate | Moderate |
| 12 | Ding S. et al. | Low | Low | Low | Low | Low | Low | Low | Low |
| 13 | Duan X. et al. | Low | Low | Low | Low | Low | Moderate | Low | Moderate |
| 14 | Gao Y. et al. | Serious | Low | Low | Low | Low | Low | Low | Serious |
| 15 | He W. et al. | Low | Low | Low | Low | Low | Low | Low | Low |
| 16 | Hu C. et al. | Serious | Low | Low | Low | Low | Low | Low | Serious |
| 17 | Jiang P. et al. | Low | Low | Low | Low | Low | Moderate | Low | Moderate |
| 18 | Liu H. et al. | Low | Low | Low | Low | Low | Moderate | Low | Moderate |
| 19 | Liu J. et al. | Moderate | Low | Low | Low | Low | Moderate | Low | Moderate |
| 20 | Wang Y. et al. | Serious | Low | Low | Low | Low | Moderate | Low | Serious |
| 21 | Yang F. et al. | Serious | Low | Low | Low | Low | Low | Low | Serious |

**Supplementary File 2 Table 3. GRADE assessment of the certainty of evidence for outcomes of outpatient pooling reforms in China**

| **GRADE Domain** | **No downgrade (0)** | **Downgrade one level (−1)** | **Downgrade two levels (−2)** |
| --- | --- | --- | --- |
| **Risk of bias** | Bias unlikely to materially alter the results. | Important limitations may lower confidence. | Major limitations substantially weaken confidence. |
| **Inconsistency** | Effects are broadly consistent, or differences are explainable. | Important unexplained differences across studies. | Large, pervasive, and unexplained differences across studies. |
| **Indirectness** | Evidence largely matches the target PICO. | Important PICO differences may modify the effect. | Evidence is substantially removed from the target PICO. |
| **Imprecision** | Information size is adequate, and the main/larger studies provide a sufficiently stable estimate of the effect. | Information size is limited, or available estimates cannot reliably distinguish an important effect from little or no effect. | Information is very sparse, and the true effect could plausibly lie across substantially different effect ranges. |
| **Publication bias** | No convincing signals. | Several signals raise concern. | Strong evidence of dissemination bias. |

**Supplementary File 2 Table4. GRADE Assessment of the Certainty of Evidence for Each Outcome**

| **No.** | **Outcome** | **Supporting studies** | **Risk of bias** | **Inconsistency** | **Indirectness** | **Imprecision** | **Publication bias** | **Certainty** |
| --- | --- | --- | --- | --- | --- | --- | --- | --- |
| 1 | Outpatient visits | 1, 3, 4, 6, 7, 11, 16, 20, 21 | Very serious | Not serious | Not serious | Not serious | Undetected | Low ●●○○ |
| 2 | Inpatient admissions | 1, 4, 5, 6, 7, 11, 15, 16, 20, 21 | Very serious | Not serious | Not serious | Not serious | Undetected | Low ●●○○ |
| 3 | Hospitalization days | 1, 2, 4, 5 | Very serious | Not serious | Serious | Not serious | Undetected | Very low ●○○○ |
| 4 | Average inpatient expenditure per admission | 1, 2, 5, 7, 11, 15, 16 | Very serious | Not serious | Not serious | Not serious | Undetected | Low ●●○○ |
| 5 | Per capita inpatient expenditure | 1, 4, 5, 6, 15 | Very serious | Not serious | Not serious | Not serious | Undetected | Low ●●○○ |
| 6 | Average outpatient expenditure per visit | 1, 7, 11, 16 | Very serious | Not serious | Not serious | Not serious | Undetected | Low ●●○○ |
| 7 | Per capita outpatient expenditure | 1, 3, 4, 6, 14 | Very serious | Serious | Not Serious | Not serious | Undetected | Very low ●○○○ |
| 8 | Total medical expenditure | 1, 4, 15 | Very serious | Serious | Serious | Not serious | Undetected | Very low ●○○○ |
| 9 | Total reimbursement expenditure | 1, 4, 7, 15, 21 | Very serious | Serious | Serious | Not serious | Undetected | Very low ●○○○ |
| 10 | Outpatient reimbursement expenditure | 4, 7, 9, 10, 21 | Very serious | Serious | Serious | Not serious | Undetected | Very low ●○○○ |
| 11 | Inpatient reimbursement expenditure | 2, 4, 5, 7, 15, 21 | Very serious | Not serious | Serious | Not serious | Undetected | Very low ●○○○ |
| 12 | Total out-of-pocket expenditure | 1, 4, 15 | Very serious | Serious | Serious | Not serious | Undetected | Very low ●○○○ |
| 13 | Outpatient out-of-pocket expenditure | 3, 6, 7, 9, 10, 14 | Very serious | Serious | Not Serious | Not serious | Undetected | Very low ●○○○ |
| 14 | Inpatient out-of-pocket expenditure | 5, 6, 7, 15 | Very serious | Not serious | Not serious | Not serious | Undetected | Low ●●○○ |
| 15 | Number of ADL limitations | 6, 18, 19 | Very serious | Not serious | Not serious | Serious | Undetected | Very low ●○○○ |
| 16 | Outpatient visitsat primary care institutions | 3, 11, 12, 13, 14, 21 | Very serious | Not serious | Not serious | Not serious | Undetected | Low ●●○○ |
| 17 | Outpatient visitsat secondary and tertiary hospitals | 3, 12, 13, 21 | Very serious | Serious | Not serious | Not serious | Undetected | Very low ●○○○ |
| 18 | Inpatient visitsat secondary and tertiary hospitals | 12, 15, 21 | Very serious | Not serious | Not serious | Not serious | Undetected | Low ●●○○ |
